# Liver fat quantification at 0.55 T enabled by locally low‐rank enforced deep learning reconstruction

**DOI:** 10.1002/mrm.70057

**Published:** 2025-08-29

**Authors:** Majd Helo, Dominik Nickel, Stephan Kannengiesser, Thomas Kuestner

**Affiliations:** ^1^ Medical Image and Data Analysis (MIDAS.lab), Department of Diagnostic and Interventional Radiology University of Tuebingen Tuebingen Germany; ^2^ Research & Clinical Translation, Magnetic Resonance, Siemens Healthineers AG Erlangen Germany

**Keywords:** deep learning, liver PDFF, locally low‐rank, low‐field MRI, reconstruction

## Abstract

**Purpose:**

The emergence of new medications for fatty liver conditions has increased the need for reliable and widely available assessment of MRI proton density fat fraction (MRI–PDFF). Whereas low‐field MRI presents a promising solution, its utilization is challenging due to the low SNR. This work aims to enhance SNR and enable precise PDFF quantification at low‐field MRI using a novel locally low‐rank deep learning–based (LLR–DL) reconstruction.

**Methods:**

LLR–DL alternates between regularized SENSE and a neural network (U‐Net) throughout several iterations, operating on complex‐valued data. The network processes the spectral projection onto singular value bases, which are computed on local patches across the echoes dimension. The output of the network is recast into the basis of the original echoes and used as a prior for the following iteration. The final echoes are processed by a multi‐echo Dixon algorithm. Two different protocols were proposed for imaging at 0.55 T. An iron‐and‐fat phantom and 10 volunteers were scanned on both 0.55 and 1.5 T systems. Linear regression, t‐statistics, and Bland–Altman analyses were conducted.

**Results:**

LLR–DL achieved significantly improved image quality compared to the conventional reconstruction technique, with a 32.7% increase in peak SNR and a 25% improvement in structural similarity index. PDFF repeatability was 2.33% in phantoms (0% to 100%) and 0.79% in vivo (3% to 18%), with narrow cross‐field strength limits of agreement below 1.67% in phantoms and 1.75% in vivo.

**Conclusion:**

An LLR–DL reconstruction was developed and investigated to enable precise PDFF quantification at 0.55 T and improve consistency with 1.5 T results.

## 1 INTRODUCTION

Proton density fat fraction (PDFF) is a quantitative imaging biomarker that allows for accurate, noninvasive assessment of liver steatosis. Utilizing chemical‐shift–encoded MRI techniques, PDFF differentiates between water and fat proton signals, providing a reliable alternative to liver biopsy and MR spectroscopy due to its lower sampling variability and practicality in clinical settings.^1^, ^2^, ^3^, ^4^, ^5^, ^6^, ^7^, ^8^, ^9^

PDFF is used in the clinical evaluation of metabolic associated fatty liver disease and has further relevance in oncology, where hepatic steatosis can serve as a predictor or manifestation of chemotherapy‐induced toxicity.^10^, ^11^ The introduction of the novel medication resmetirom to treat noncirrhotic metabolic‐associated steatohepatitis has increased the need for regular monitoring of liver fat content.^12^, ^13^ Furthermore, the apparent transverse relaxation rate $R_{2}^{*}$ is an established biomarker for iron overload quantification in the liver and can be simultaneously obtained with the same MRI technique. Liver $R_{2}^{*}$ is directly correlated to total body iron stores,^14^ making liver iron concentration a suitable measure for assessing overall body iron levels.^15^, ^16^ Whereas high‐field MRI has traditionally been preferred for its superior SNR and spatial resolution, recent advancements in hardware and software have greatly improved the performance of low‐field MRI systems. Low‐field MRI is particularly beneficial for imaging near metallic implants due to reduced susceptibility artifacts^17^ and has shown promise in lung imaging, including persistent pulmonary damage in COVID‐19 patients^18^ and many different applications that underscore the growing role of low‐field MRI in clinical practice.^19^, ^20^, ^21^, ^22^, ^23^, ^24^, ^25^, ^26^, ^27^

Ongoing research continues to refine low‐field MRI potential and expand its applications. However, water–fat separation—needed for PDFF estimation—is a challenging task because the chemical shift decreases with lower magnetic field strength.^28^ PDFF is a well‐established technique that is extensively tested and offered by various vendors on 1.5 and 3 T MRI systems.^29^ Given its sensitivity to SNR and the intrinsically prolonged scan times compared to a single echo acquisition, it still needs to be established for 0.55 T. A recent study^30^ employed a Monte Carlo simulation to optimize acquisition protocol parameters for 0.55 T and investigated advanced locally low‐rank (LLR) denoising methods, namely robust LLR and random matrix theory, to address noise bias in PDFF measurements. Robust LLR estimates noise levels using Gaussian‐distributed random matrices,^31^ and the singular value threshold can be calculated using a Stein's unbiased risk estimate–based approach.^32^, ^33^ Random matrix theory–based denoising estimates noise levels and eliminates noise components by utilizing the spectral characteristics of random Gaussian matrices^34^ and has been particularly effective in diffusion MRI due to the availability of multiple contrasts.^35^

Using advanced reconstruction techniques is essential to (i) accelerate the acquisition, and (ii) enhance image quality, especially in the case of PDFF, where noisy echo images might lead to noise bias or local water–fat swaps. Recent advancements in deep learning (DL) have transformed MRI reconstruction by achieving significant improvements in both temporal and spatial resolution. DL‐based approaches leverage multi‐coil data and incorporate advanced image‐domain regularization and k‐space interpolation, improving upon traditional acceleration techniques.^36^, ^37^ Physics‐driven DL methods further enhance reconstruction quality by integrating MRI physics into learning models, ensuring data consistency with the measured k‐space while addressing challenges such as complex‐valued data and nonlinear forward models.^38^, ^39^

In this work, an iterative LLR–DL reconstruction method is proposed to enhance image quality on 0.55 T systems and enable precise PDFF and $R_{2}^{*}$ quantification using a multi‐echo gradient echo (GRE) sequence within an acquisition time of a clinically acceptable breath hold. Two scan protocols with different resolution are proposed, and their precision to measure PDFF in vivo and in vitro was investigated.

## 2 METHODS

### 2.1 Multi‐echo water–fat separation

Multi‐echo techniques for chemical shift–based water–fat separation have experienced increased usage in the clinical routine. PDFF uses a GRE sequence with a low flip angle (FA) to minimize T1 bias. Commonly, multiple echoes are acquired at *TE*_*n*_.^7^, ^40^ The acquired signal *M*_*n*_ is given by:

(1) $$M_{n}=\left(M_{w}\;e^{-R_{2w}^{*}{\mathrm{TE}}_{n}}+C_{n}M_{f}\;e^{-R_{2f}^{*}{\mathrm{TE}}_{n}}\right)E_{n},$$

where *M*_*w*_ and *M*_*f*_ are the water and fat signals. $R_{2w}^{*}$ and $R_{2f}^{*}$ are the relaxation rates for the respective signals. *E*_*n*_ is a complex phasor that accounts for phase errors caused by systematic imperfections, including off‐resonance effects, eddy currents, and gradient delays. *C*_*n*_ is a complex coefficient that models a precalibrated fat spectrum and can be computed using:

(2) $$C_{n}=\sum_{i=1}^{m}w_{i}e^{2\pi ∆f_{i}{\mathrm{TE}}_{n}},$$

with *m* being the number of the spectral fat peaks, *w*_*i*_ the relative amplitudes of the fat peaks, and *∆f*_*i*_ the chemical shifts between fat and water protons. The sum of the weighting factors *w*_*i*_ is normalized to 1.0. There are different precalibrated fat spectra in the literature,^15^, ^40^, ^41^, ^42^ including a model that only uses the main fat peak at −3.3 ppm. The fat signal should, however, be modeled as a combination of several frequency components because fat is well known to have multiple spectral peaks.^43^, ^44^, ^45^, ^46^, ^47^, ^48^, ^49^, ^50^, ^51^, ^52^

By taking the magnitude of Equation (1), the term *E*_*n*_ is eliminated. Furthermore, the terms $R_{2w}^{*}$ and $R_{2f}^{*}$ are combined into a single relaxation rate $R_{2\mathrm{eff}}^{*}$ for the water–fat mixture. The signal model can be written as:

(3) $$\left|M_{n}\right|=\left|\left(M_{w}+C_{n}M_{f}\;\right)e^{-R_{2\mathrm{eff}}^{*}{\mathrm{TE}}_{n}}\right|.$$

This simplification reduces the complexity of the equation and enhances the numerical stability of the solution.^53^ For n≥3, the equation can be solved using a nonlinear least squares fitting algorithm. In this study, we utilize the nine peaks fat model^42^, ^54^ and multi‐step adaptive fitting with the Levenberg–Marquardt algorithm.^55^ To obtain accurate initial estimates for *M*_*w*_ and *M*_*f*_ with preserved phase information and reduced water–fat ambiguity, a water–fat separation is performed using the complex‐based two‐point Dixon method. $R_{2}^{*}$ is initialized with a constant value, for example, 30*s* ^−1^. The PDFF map in % is computed using:

(4) $$\text{PDFF}=\frac{M_{f}}{M_{f}+M_{w}}.$$

### 2.2 PDFF at low‐field

At low‐field, tissues experience shorter $T_{1}$relaxation time and prolonged $T_{2}$ and $T_{2}^{*}$ relaxation times.^28^ Water–fat separation becomes more challenging due to the reduced chemical shift between water and fat. Specifically, the frequency difference between water and the main methylene (CH_2_) fat peak is approximately 80 Hz at 0.55 T, compared to about 220 Hz at 1.5 T. This smaller spectral separation leads to a longer in‐phase and opposed‐phase cycle, making conventional approaches that acquire multiple in‐phase and opposed‐phase contrasts impractical because they would require a TR of around 35 ms, which would significantly prolong the acquisition time. To enable accurate PDFF quantification under these constraints, a multi‐echo GRE acquisition with equidistant echoes and minimal TE spacing is preferred. This approach still provides sufficient sampling of the water–fat signal evolution while maintaining a reasonable scan time. However, to initialize *M*_*w*_ and *M*_*f*_ for the two‐point Dixon method, it is crucial to choose two echoes that are acquired near in‐phase and opposed‐phase TEs, that is, TE_1_ = 4 to 5 ms and TE_2_ = 8 to 10 ms. Otherwise, local water–fat swaps would occur not only in the initial guess but also in the fitted model for PDFF. A detailed overview of the suggested scan protocols is given in section 2.5.

### 2.3 Locally low‐rank enforced deep learning reconstruction

In this work, an iterative DL reconstruction technique based on regularized SENSE^56^ is proposed. The method alternates between regularized SENSE and the estimation of a better prior using a neural network, where the best regularization setting is learned during training. For iteration i, the optimization problem can be written as follows:

(5) $${\hat{m}}_{i+1}={\text{argmin}}_{m}\left({\left\|\mathrm{Sm}-a\right\|}^{2}+\frac{1}{\lambda_{i}^{2}}\;{\left\|m-z_{i}\right\|}^{2}\right),$$

where ${\hat{m}}_{i+1}$ is the reconstructed image in each iteration, S is the coil sensitivity encoding operator that multiplies the estimated image **m** with the coil images and superimposes the result according to the acceleration pattern, and a denotes the aliased coil images. Furthermore, $\lambda_{i}$ and $z_{i}$ are the regularization factor and the prior in each iteration, respectively. This formulation corresponds to a proximal mapping of the data consistency term, where the regularization enforces proximity to a learned prior.^57^, ^58^, ^59^ To solve this optimization problem, a singular value decomposition $S=U\Sigma V^{†}$ can be computed:

(6) $${\hat{m}}_{i+1}=V\;\frac{1}{1+\lambda_{i}^{2}\Sigma^{2}}V^{†}\left(\lambda_{i}^{2}\;S^{†}a+z_{i}\;\right).$$

#### 2.3.1 Low‐rank property

For simplicity, the iteration index i is omitted in the following equations. Let ${\hat{m}}_{E,N}$ be the signal matrix that contains all E echoes and N voxels. To exploit the spectral sparsity, $N_{x}$ rows containing E elements are extracted from $\hat{m}$ using a reshape operator ${ℬ}_{x}:\left(\;{\hat{m}}_{E,N}\to {\hat{m}}_{E,N_{x}}\;\right)$.^31^ These rows correspond to local image patches with $\left(E\ll N_{x}\ll N\right)$. Using the singular value decomposition:

(7) $${\hat{m}}_{E,N_{x}}=U_{E,r}^{\left(x\right)}\;\Sigma_{r,\;r}^{\left(x\right)}\;V_{r,N_{x}}^{\left(x\right)†}.$$

The upper index (*x*) denotes the separate decomposition for each spatial position with processing by a patch size of 1 × 3 × 3. $U_{E,r}^{\left(x\right)}$ represents the local bases of the spectral components and can be used to project the reconstructed images to the spectral contributions of water and fat:

(8) $$P_{N,\;r}^{\left(x\right)}=\sum_{E}U_{E,r}^{\left(x\right)}{\hat{m}}_{E,N},$$

where a strong order in signal intensities is anticipated along the spectral dimension *r*. Smaller singular values tend to carry most of the noise. Previous studies have focused on identifying the optimal cutoff for these values to enhance denoising.^31^, ^32^ In this work, a different approach is taken by using a neural network to jointly denoise all image projections across the singular value basis.

The above projection $U=U_{E,r}^{\left(x\right)}$ can be integrated into the iterative reconstruction scheme by setting $z_{i}=U^{†}\;f_{i}\left(U\;{\hat{m}}_{i-1}\right)$ with $z_{0}=0$; $f_{i}$ is a trainable neural network operating on the low‐rank patches. Consequently, a joint regularization of all echoes is achieved, exploiting the correlation along the echo dimension and operating on the decomposed spectral components.

**FIGURE 1:**
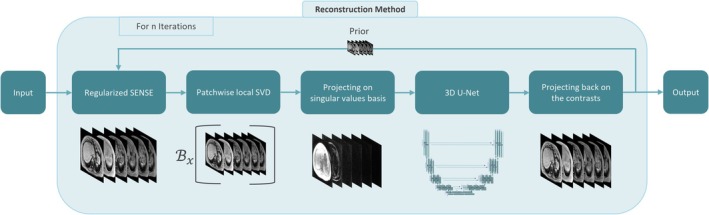
Visualization of the proposed LLR–DL reconstruction method. The echo images are reconstructed using regularized SENSE with patch‐wise local SVD being performed along the echo dimension. The echoes are projected on their singular values and processed using U‐Net architecture. The low‐rank output of the U‐Net is projected on the original images and used as prior for subsequent iterations. DL, deep learning; LLR, locally low‐rank; SVD, singular value decomposition.

An overview of the entire pipeline is depicted in Figure 1. Regularized SENSE is performed on the acquired contrasts. The images are projected on their singular value basis using a precalculated projection matrix (Equation 7). The projections are inserted into a U‐Net,^60^ and the output of the network is projected back on the original images. The final output is given as prior for regularized SENSE in the next iteration. This process is performed n times, and the output of the reconstruction pipeline is used to perform water–fat separation using the multi‐step adaptive fitting method to produce PDFF and $R_{2}^{*}$ maps.

In summary, this approach is based on the standard regularized SENSE formulation as described in Equation 5 and incorporates a U‐Net operating on low‐rank images to compute the prior by exploiting spatiospectral correlations inherent in multi‐echo acquisitions. In the context of six‐echo acquisitions designed to model both water and fat components, the signal exhibits spectral sparsity, dominated by two primary spectral components. As a result, the underlying data matrix has an effective rank of two, making it well suited for low‐rank modeling. The reconstruction framework relies on a physical signal model that enforces data consistency, similar to compressed sensing.^61^ The network focuses on local image enhancement and is designed to be largely independent of the specific image content, which is crucial for ensuring generalizability across different clinical conditions. Recent studies^59^, ^62^ have shown that, when trained on sufficiently broad datasets, neural networks generalize well and are mainly sensitive to SNR rather than to anatomical variations. This supports the robustness of our approach and minimizes dependency on the data distribution.

### 2.4 Data for training

For training the network, a total of 48 fully sampled datasets with a matrix size of 256 × 224 × 64 were acquired on 1.5 and 3 T MRI systems (MAGNETOM scanners, Siemens Healthineers, Forchheim, Germany) using a 3D multi‐echo GRE sequence with a TR of 9.0 ms. Data were collected during free‐breathing in the brain and pelvis of healthy volunteers without medical assessment. These anatomical regions were selected because they permit fully sampled acquisitions with minimal motion artifacts, which would not be achievable in abdominal organs. Additionally, a pretrained model was employed that had been trained on SPACE (sampling perfection with application optimized contrasts using different flip angle evolution) and VIBE (volumetric interpolated breath‐hold examination) datasets from healthy volunteers, comprising approximately 500 fully sampled volumes, to further enhance reconstruction performance and generalization. All volunteers received an informed consent discussion and provided written consent for the further use and processing of their data. MR measurements were conducted in accordance with the Declaration of Helsinki.

The training datasets were retrospectively undersampled with varying patterns for a total acceleration factor of 4. To address memory constraints during training, the data were cropped to 1584 volumes of size 120 × 120 × 32. The dataset was split such that 60% was used for training, 20% for validation, and 20% for testing.

The number of alternating steps between SENSE and low‐rank neural network regularizers was set to n=6. A U‐Net architecture with three stages was used. A base number of 32 features, convolutional layers, leaky rectified linear unit ^63^ activation function, three average pooling layer, and transposed convolution layers and with no dropout or normalization layers were chosen.

The reconstruction network was trained in a supervised fashion utilizing an L1‐loss function, the Adam optimizer, a batch size of 1, and a learning rate of 10^−4^. Hyperparameter optimization was performed on the validation set. Additionally, the network leveraged a pretrained model trained on a large pool of turbo spin echo; in‐phase and opposed‐phase data were used. The network performance was evaluated retrospectively on the testing set using structural similarity index (SSIM)^64^ and peak SNR (PSNR) metrics. Additionally, a 3D U‐Net regularizer was trained to enable a direct performance comparison between the proposed iterative method and an established denoiser. The 3D U‐Net shares the same architecture as the regularizer used within a single iteration of the proposed framework. This network is trained in an unrolled manner and operates on the projections onto the singular value bases, effectively forming an LLR‐based denoiser. This comparison was performed solely to assess reconstruction quality and will not be included in the prospective repeatability and reproducibility study. The network was implemented in PyTorch and trained using one GPU node on an NVIDIA DGX A100 (8 GPUs each with 40GB memory and a bandwidth of 1555 GB/sec).

### 2.5 Prospective study

The reconstruction pipeline was integrated into the scanner and evaluated in a prospective study. For this study, one routine 0.55 T scanner (MAGNETOM Free.Max, Siemens Healthineers, Forchheim, Germany) and one routine 1.5 T scanner (MAGNETOM Sola, Siemens Healthineers, Forchheim, Germany) were used. A total of four acquisition protocols were implemented, with two distinct protocols designed for each field strength. The study included both phantom and volunteer data, with volunteers recruited without any prior medical assessment for fatty liver or iron depositions. The volunteer data used for this evaluation were acquired under the same ethical approvals and informed consent procedures as described for the training datasets. All participants provided written informed consent for the use of their data in research, in accordance with the Declaration of Helsinki and relevant national and European regulations.

#### 2.5.1 Acquisition parameters

**FIGURE 2:**
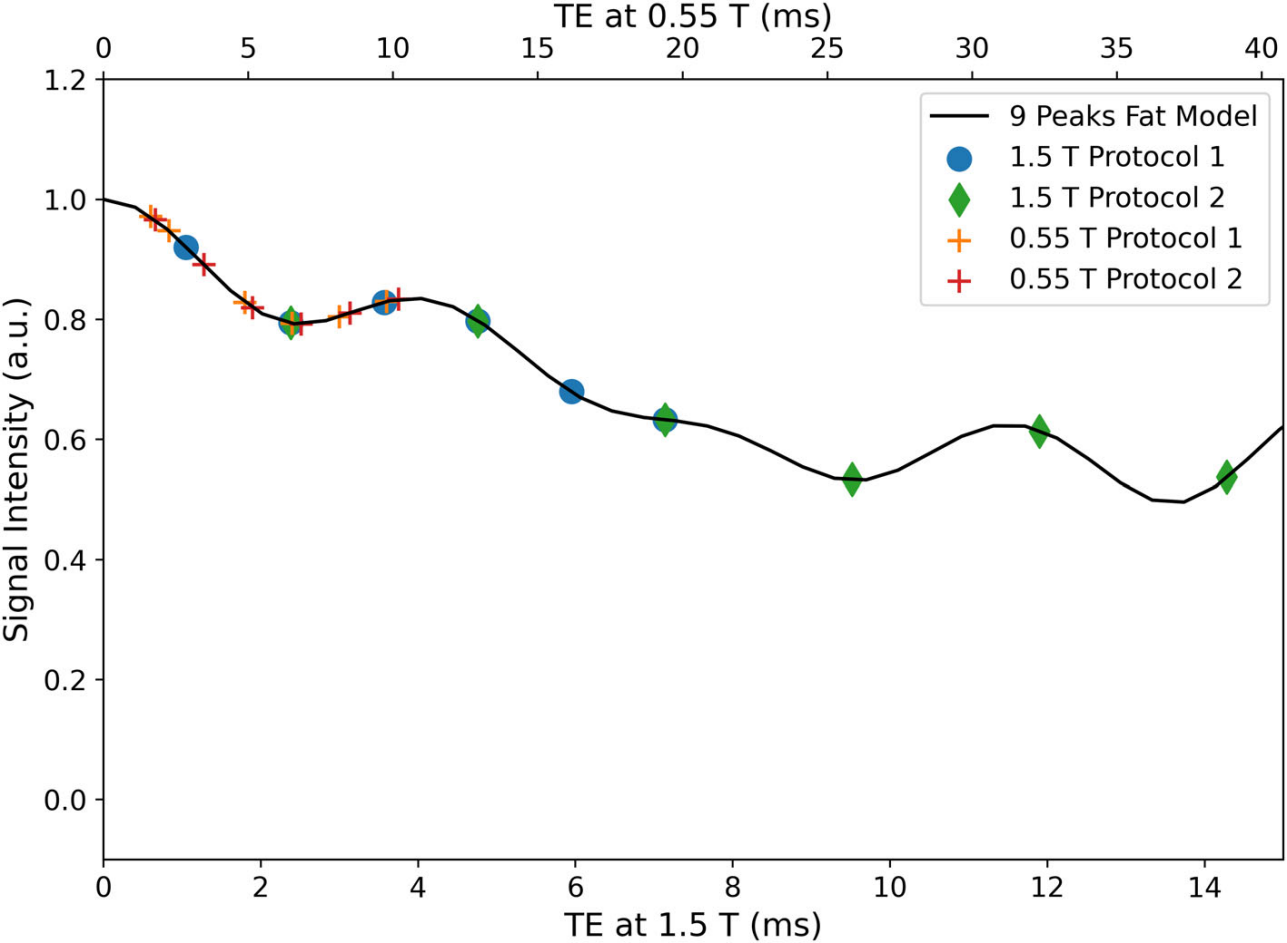
A visualization of the magnitude of the fat dephasing coefficients computed using the nine peaks fat model. The acquired TEs at the different field strengths are depicted. At 0.55 T, the echoes are acquired at minimum TE (top x‐axis) and can only sample one fat dephasing cycle, whereas it is possible to sample up to three fat dephasing cycles at 1.5 T (bottom x‐axis)

Typical PDFF scans acquire six GRE echoes at in‐phase and opposed‐phase TEs. At low‐field, six echoes are acquired at minimum TE. On 1.5 T, two vendor product protocols are used. The first protocol has an acceleration factor of R = 3 and acquires echoes at the minimum TE. The second protocol uses an acceleration factor of R = 4, with images captured at both in‐phase and opposed‐phase TEs. Both protocols acquire data with a matrix size of 160 × 140 × 72. A standard combination of build‐in spine coil matrix and 18‐channel anterior body matrix was used for the scan at 1.5 T. At low‐field, the first protocol is configured with an acceleration factor of R = 4 and a matrix size of 160 × 140 × 72, whereas the second protocol uses R = 3 with a matrix size of 128 × 112 × 72. Although larger FAs might be feasible due to the shorter T1 relaxation of water, a conservative FA of 4° was chosen to ensure consistency across field‐strengths. A flexible body coil with 24 receive elements (Contour L, Siemens Healthineers, Forchheim, Germany) was used for all scans. A comprehensive overview of the scan protocols is provided in Table 1. Additionally, Figure 2 illustrates the employed TEs of the respective acquisition protocols alongside the magnitude of the fat dephasing coefficients $C_{n}$ based on the nine‐peaks spectral model. The technical difference of the low‐field acquisition is that only one fat dephasing cycle can be sampled, whereas at high‐field, up to three fat dephasing cycles can be acquired.

**TABLE 1:** Detailed overview of the proposed acquisition protocols at 0.55 T and the vendor product protocols at 1.5 T.

Acquisition parameters	Protocol 1	Protocol 2	Protocol 1	Protocol 2
Field strength [T]	0.55	0.55	1.5	1.5
FOV [mm^2^]	350 × 400	350 × 400	350 × 400	350 × 400
Acquired matrix size	109 × 160 × 48	89 × 128 × 48	111 × 160 × 48	109 × 160 × 44
Reconstructed matrix size (with slice oversampling)	140 × 160 × 72 (96)	112 × 128 × 72 (96)	140 × 160 × 72 (96)	140 × 160 × 72 (88)
Slice sickness (reconstructed/acquired) [mm]	3.0/6.0	3.0/6.0	3.0/6.0	3.5/7.0
PAT acceleration	4	3	3	4
Number of echoes	6	6	6	6
Flip angle [degree°]	4	4	4	4
TR [ms]	12.5	11.8	9.2	15.6
TE [ms]	1.8, 3.4, 5.1, 6.8, 8.5, 10.2	1.6, 3.3, 4.9, 6.5, 8.2, 9.8	1.3, 2.6, 3.9, 5.2, 6.5, 7.8	2.4, 4.8, 7.1, 9.5, 11.9, 14.3
Scan time [s]	16.8	17.2	16.2	18.9

The datasets were reconstructed using conventional parallel imaging, specifically controlled aliasing in parallel imaging results in higher acceleration (CAIPIRINHA)^65^ and the proposed DL reconstruction method.

#### 2.5.2 Phantom repeatability and reproducibility

**FIGURE 3:**
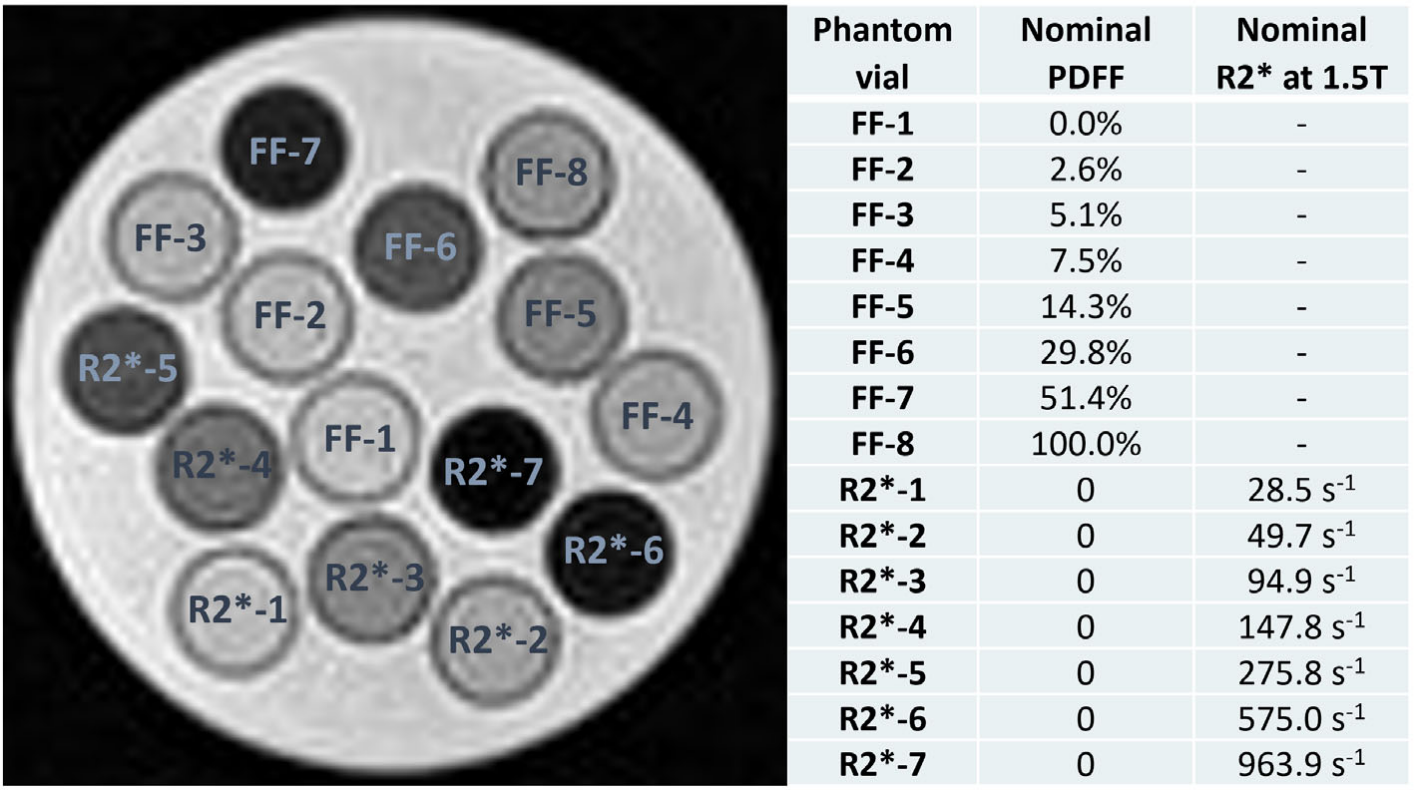
Echo image of the fat‐and‐iron phantom acquired at 0.55 T and TE = 6.8 ms (left). Nominal PDFF values of eight vials from the datasheet and $R_{2}^{*}$ values at 1.5 T (right). PDFF, proton density fat fraction.

In this study, a fat‐and‐iron phantom (Calimetrix, Madison, WI) was used. Although the phantom does not have a specific model number, it is similar in design to the Calimetrix Model 450. This phantom is specifically designed for validating $R_{2}^{*}$ and fat quantification in MRI. It consists of a spherical acrylic housing that contains 15 cylindrical vials immersed in a water bath. The phantom housing is designed to fit a range of MR coils.^66^ Figure 3 shows an echo image at TE = 6.8 ms of the phantom acquired on 0.55 T system. The MR‐verified PDFF values of the vials marked as FF (fat fraction) in the figure are obtained from the phantom datasheet.^66^ Additionally, the nominal $R_{2}^{*}$ values at 1.5 T are presented. Peanut oil is used inside the vials to modulate PDFF because its proton NMR spectrum closely resembles that of triglyceride protons in human tissue, making it an effective simulation of liver fat.^67^, ^68^ Whereas the fat spectrum remains unaffected by temperature, the chemical shift between water and fat is temperature‐dependent. To account for this, a temperature‐corrected nine‐peak model with an offset of –0.01 ppm/°C (from 37°C) is used for the phantom data as suggested in Ref. 69, 70

The phantom was placed in the scanner room 2 h prior to the first measurement to maintain a consistent temperature of 22°C during all experiments. It was positioned in the scanner with the vials oriented in the direction of the main magnetic field and transversal slices being measured.

The scan was repeated four times, with the phantom being repositioned before each repetition. Due to sufficient SNR in the phantom data, all datasets are reconstructed using a conventional reconstruction. Regions of interest (ROIs) were defined on the middle slice of the PDFF maps around each vial for every dataset, and the mean PDFF values were recorded.

The PDFF accuracy of all four acquisitions was evaluated by calculating its linearity and bias with respect to reference values of the manufacturer. As a measure for linearity, Pearson's linear correlation coefficient *r* and its 95% confidence intervals (CIs) were used. Additionally, a linear regression analysis including intercept, slope, and the corresponding 95% CIs was performed. T‐statistics were performed on the mean differences, and a significance level *p* < 0.05 was considered statistically significant.

Bland–Altman plots were used to assess the reproducibility of the measurement, that is, bias and limits of agreement (LoA) between the different field strengths as well as between each field strength and the reference values provided by the phantom manufacturer.

#### 2.5.3 In vivo repeatability and reproducibility

To assess the repeatability and reproducibility of the method in vivo, 10 volunteers (six males and four females, age: 49.9 ± 17.6 years, weight: 76.8 ± 12.7 kg, height: 174.7 ± 9.8 cm) were scanned within 1 h on both field strengths 1.5 and 0.55 T. The scan protocols were repeated on each system, with the volunteer standing up and being repositioned on the table between scans. Bland–Altman analysis was performed to compare the PDFF values obtained from both the LLR–DL and the conventional reconstruction, using the PDFF values from the conventionally reconstructed 1.5 T PDFF maps as the reference.

In this study, volumetric evaluation of PDFF values in the liver was performed based on a liver segmentation network^71^ applied to water images and vessel exclusion using a Frangi filter^72^ applied to $R_{2}^{*}$ maps.^73^ All statistical analysis is performed in Python using SciPy and NumPy.

## 3 RESULTS

### 3.1 Reconstruction results

On the retrospective test dataset, the proposed LLR–DL framework has achieved images with PSNR=35.93±2.90dB and SSIM=0.95±0.02, whereas the 3D U‐Net regularizer yielded images with PSNR=32.60±2.70dB and SSIM=0.92±0.02. By comparison, the conventional reconstruction technique produced images with PSNR=27.06±3.53dB and SSIM=0.76±0.12. The proposed LLR–DL framework outperformed both the 3D U‐Net denoiser and the conventional SENSE‐only reconstruction, demonstrating the benefit of integrating learned regularization within a data‐consistent iterative model. For visual comparison, Figures S1–S3 have been added, showing reconstructed images from different methods. Three representative liver cases with low PDFF values of 4.5% (Figure S1) and 5% (Figure S2), and a midrange PDFF of 10% (Figure S3), are included to illustrate reconstruction performance across a range of fat content.

**FIGURE 4:**
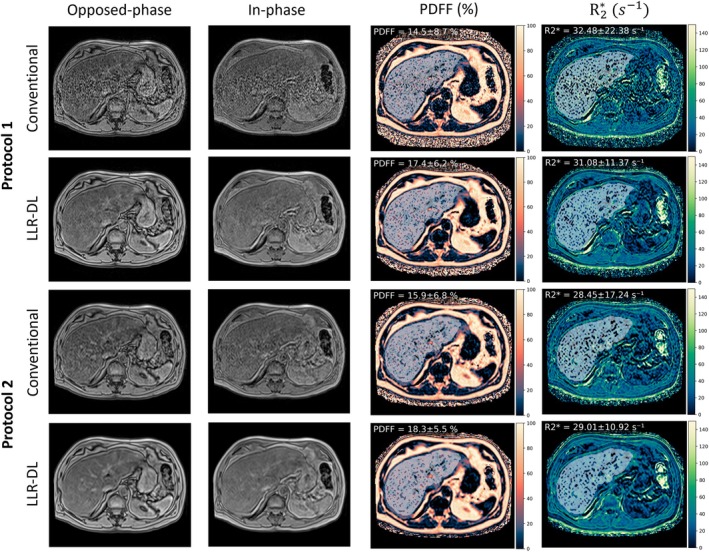
Reconstruction results of opposed‐phase and in‐phase images at low‐field corresponding to echoes 3 and 6 in both protocols. The upper two rows are the reconstruction results of the data acquired using protocol 1. The proposed LLR–DL shows superior image quality to the conventional CAIPIRINHA reconstruction. The lower two rows show the reconstruction results of protocol 2. Here, the conventional CAIPIRINHA method provides images with good SNR; however, the LLR–DL enhanced the SNR. For all reconstruction results, the SNR level is visible in the fitted quantitative PDFF and $R_{2}^{*}$ Maps. An example ROI from the liver segmentation is overlaid on the quantitative maps, and the corresponding mean and standard deviation are reported. The corresponding high‐field dataset is shown in Figure 5, enabling cross‐field comparison of reconstruction quality. CAIPIRINHA, controlled aliasing in parallel imaging results in higher acceleration; ROI, region of interest.

**FIGURE 5:**
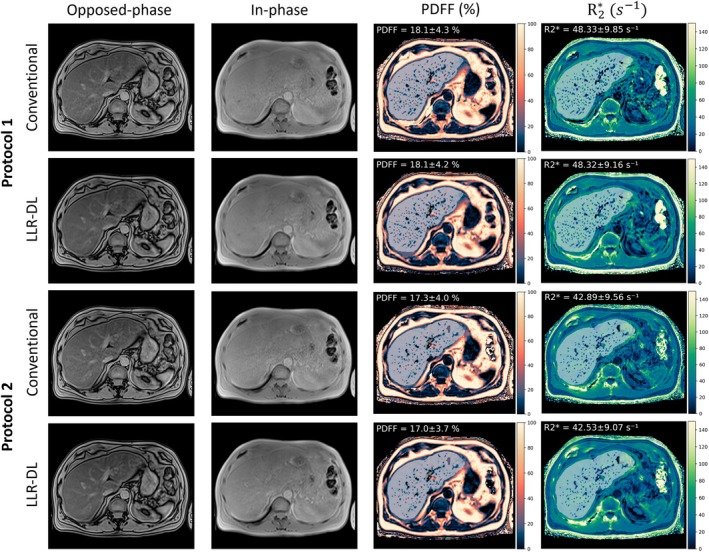
Reconstruction results of opposed‐phase and in‐phase images at 1.5 T, corresponding to echoes 2 and 4 (protocol 1) and echoes 1 and 2 (protocol 2). The upper two rows are the reconstruction results of the data acquired using protocol 1. The lower two rows show the reconstruction results of protocol 2. Both LLR–DL and conventional CAIPIRIHINIA provided images with good SNR. This dataset is acquired on the same volunteer as in Figure 4. An example ROI from the liver segmentation is overlaid on the quantitative maps, and the corresponding mean and standard deviation are reported.

The prospective dataset acquired at low‐field using different protocols was reconstructed with both conventional and LLR–DL. Figure 4 presents a visual comparison between these reconstruction techniques. The echoes used for the two‐point Dixon method for initial water and fat separation are shown along with the final PDFF and $R_{2}^{*}$ maps. An example ROI from the liver segmentation is overlaid on the images, and the corresponding mean and standard deviation of the PDFF and $R_{2}^{*}$ values within the ROI are reported. The reconstructed images using conventional reconstruction show low SNR for protocol 1, whereas protocol 2 demonstrates significantly better SNR due to its lower acceleration factor and smaller matrix size. However, noise still affects the fitting results and is evident in the quantitative PDFF and $R_{2}^{*}$ maps for both protocols. The proposed LLR–DL reconstruction significantly enhances the image quality, leading to higher SNR in the acquired contrasts. This improvement in SNR is also reflected in the quantitative maps, demonstrating the superiority of the proposed technique. Please refer to Figure S4 for a zoomed‐out view of a manually drawn ROI in the liver. Figure 5 shows the corresponding high‐field dataset reconstructed using CAIPIRINHA and LLR–DL, enabling a cross‐field comparison of image quality. LLR–DL did not exhibit a noticeable advantage because CAIPIRINHA already delivers images with high SNR.

### 3.2 Phantom repeatability and reproducibility

The regression analysis of the in vitro experiments (Table 2) demonstrated (all values in PDFF %) a slope of 1.00 with 95% CI [0.97,1.02] for both protocols on 0.55 T and a slope of 1.01 with the 95% CI [0.99,1.04] and slope of 1.01 with the 95% CI [0.98,1.04] for protocols 1 and 2 on 1.5 T. Intercept CIs were reported as follows: for 0.55 T, the intervals were −1.14 [−2.13, −0.15] in protocol 1 and −1.19 [−2.20, −0.18] in protocol 2. Higher values were observed at 1.5 T, with −1.47 [−2.58, −0.36] for protocol 1 and −1.29 [−2.49, −0.09] for protocol 2. The linear correlation between the measured PDFF and reference phantom values reveal an exceptionally high Pearson correlation coefficient (r = 1.0) and a (*p*‐value <0.01), indicating a perfect linear relationship with statistically significant correlation. The average range (precision) quantifies measurement variability by calculating the average of peak‐to‐peak differences (maximum – minimum) across repetitions for each ROI. Precision values of 1.55 and 1.48 for protocol 1 and 2 at 0.55 T and 0.83 and 1.15 for protocol 1 and 2 at 1.5 T were found. For a visual interpretation of the linear regression results, please refer to Figure S5.

**TABLE 2:** Linear regression analysis comparing ROI‐based MRI–PDFF measurements with reference values from the phantom datasheet.

	Slope (95% CI)	Intercept (95% CI)	*r* (95% CI)	Average range
0.55 T protocol 1	1.00 [0.97,1.02]	−1.14 [−2.13, −0.15]	1.00 [1.00, 1.00]	1.55
0.55 T protocol 2	1.00 [0.97, 1.02]	−1.19 [−2.20, −0.18]	1.00 [1.00, 1.00]	1.48
1.5 T protocol 1	1.01 [0.99, 1.04]	−1.47 [−2.58, −0.36]	1.00 [0.99, 1.00]	0.83
1.5 T protocol 2	1.01 [0.98, 1.04]	−1.29 [−2.49, −0.09]	1.00 [0.99, 1.00]	1.15

*Note*: The table summarizes the slope and intercept of the regression lines, along with the Pearson correlation coefficient *r* and their respective 95% CIs for each protocol and field strength.

Abbreviations: CI, confidence interval; PDFF, proton density fat fraction; ROI, region of interest.

Table 3 presents the repeatability coefficients of the four repetitions in each protocol. At 0.55 T, the repeatability coefficients in PDFF units were 2.33% for protocol 1 and 2.28% for protocol 2. At 1.5 T, the coefficients were 1.60% for protocol 1 and 1.84% for protocol 2. At 0.55 T, the t‐statistics are 1.35 for protocol 1 and 1.25 for protocol 2. At 1.5 T, the t‐statistics are 1.62 for both protocol 1 and protocol 2. Most important, for all protocols, the *p*‐values were greater than 0.05, indicating that there were no statistically significant differences between the measured PDFF values and the reference phantom values.

**TABLE 3:** Repeatability coefficients in PDFF units of the in vitro phantom measurements in addition to the t‐statistics analysis.

	Repeatability coefficient	t‐statistic	*p*‐value
0.55 T protocol 1	2.33%	1.35	0.22
0.55 T protocol 2	2.28%	1.25	0.25
1.5 T protocol 1	1.60%	1.62	0.15
1.5 T protocol 2	1.84%	1.62	0.15

*Note*: No significant difference between the measured and nominal PDFF values for *p*‐value <0.05 is observed.

**FIGURE 6:**
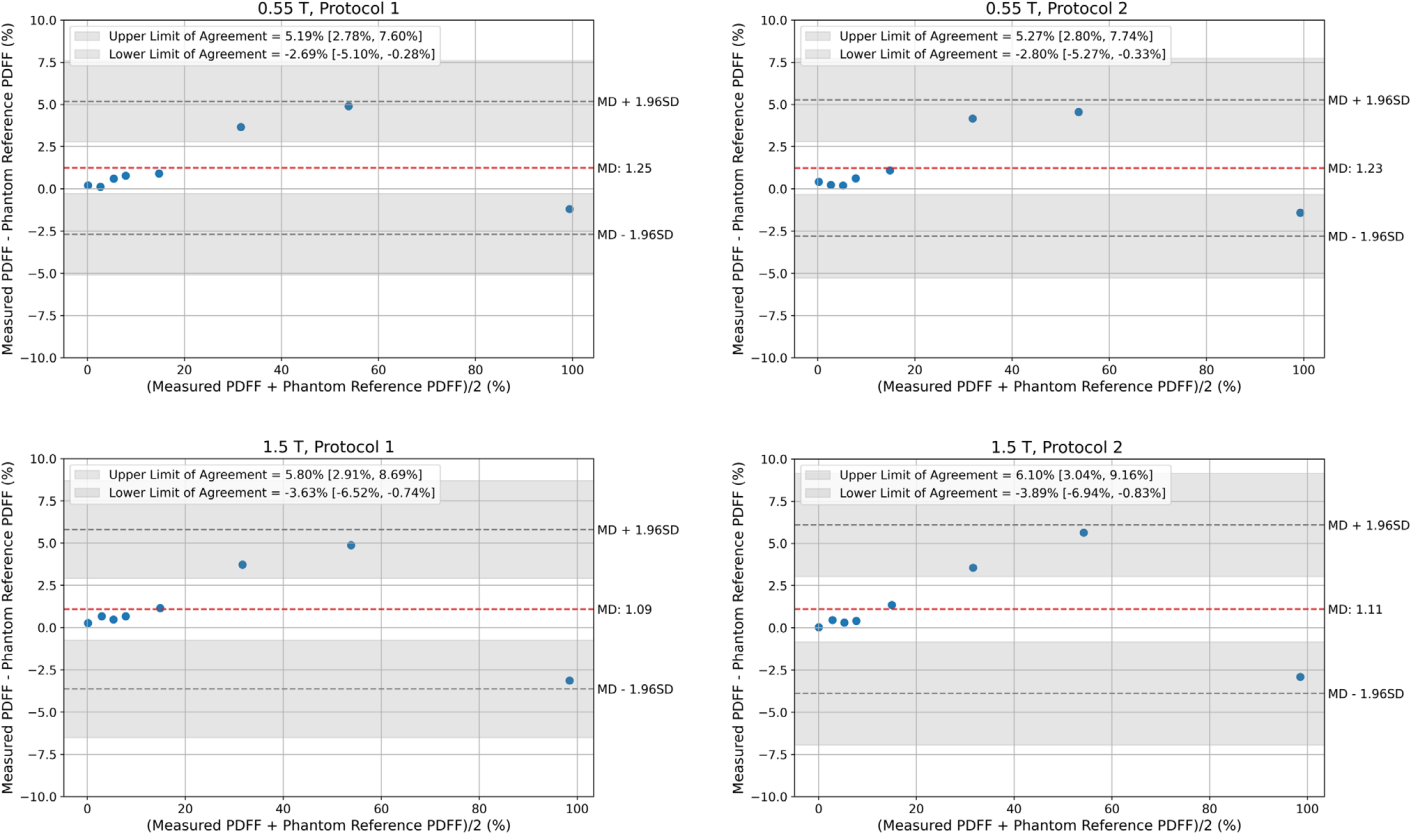
PDFF assessment of phantom. The upper row shows the measurements at 0.55 T and the lower row shows the measurements at 1.5 T. The mean of the scan repetitions was used to compute these Bland–Altman plots. The MD values of the different protocols ranged between 1.09 and 1.25. Similar LoA were achieved using the different protocols. MD, mean difference. LoA, limits of agreement.

The Bland–Altman plots in Figure 6 compare the measured PDFF averaged over the repetitions for all protocols to the reference values from the phantom. At 0.55 T, protocol 1 and 2 show very similar mean difference (bias) of 1.25% and 1.23%, respectively. At 1.5 T, protocol 1 and 2 have mean difference of 1.09% and 1.11%, respectively. A strong agreement across field strengths was observed, with the reproducibility coefficient (defined as the 95% LoA) reaching its worst value for the comparison between 0.55 T protocol 2 and 1.5 T protocol 2, at 1.67% [0.98%, 2.36%] with a mean difference of 0.54%.

### 3.3 In vivo repeatability and reproducibility

The liver segmentation with vessel segmentation was run on all data. Although the liver segmentation worked well on all data, the vessel segmentation was not accurate on the 0.55 T datasets for the conventionally reconstructed images due to the low SNR in the $R_{2}^{*}$ maps. Further details on the results of liver segmentation and vessel exclusion are not presented because this is not the main focus of this work. The segmentation masks computed on the LLR–DL reconstructed images are also used for the computation of mean PDFF of the conventional reconstructed images to minimize bias due to segmentation. Furthermore, this ensures that the exact same ROIs are used for the datasets.

**FIGURE 7:**
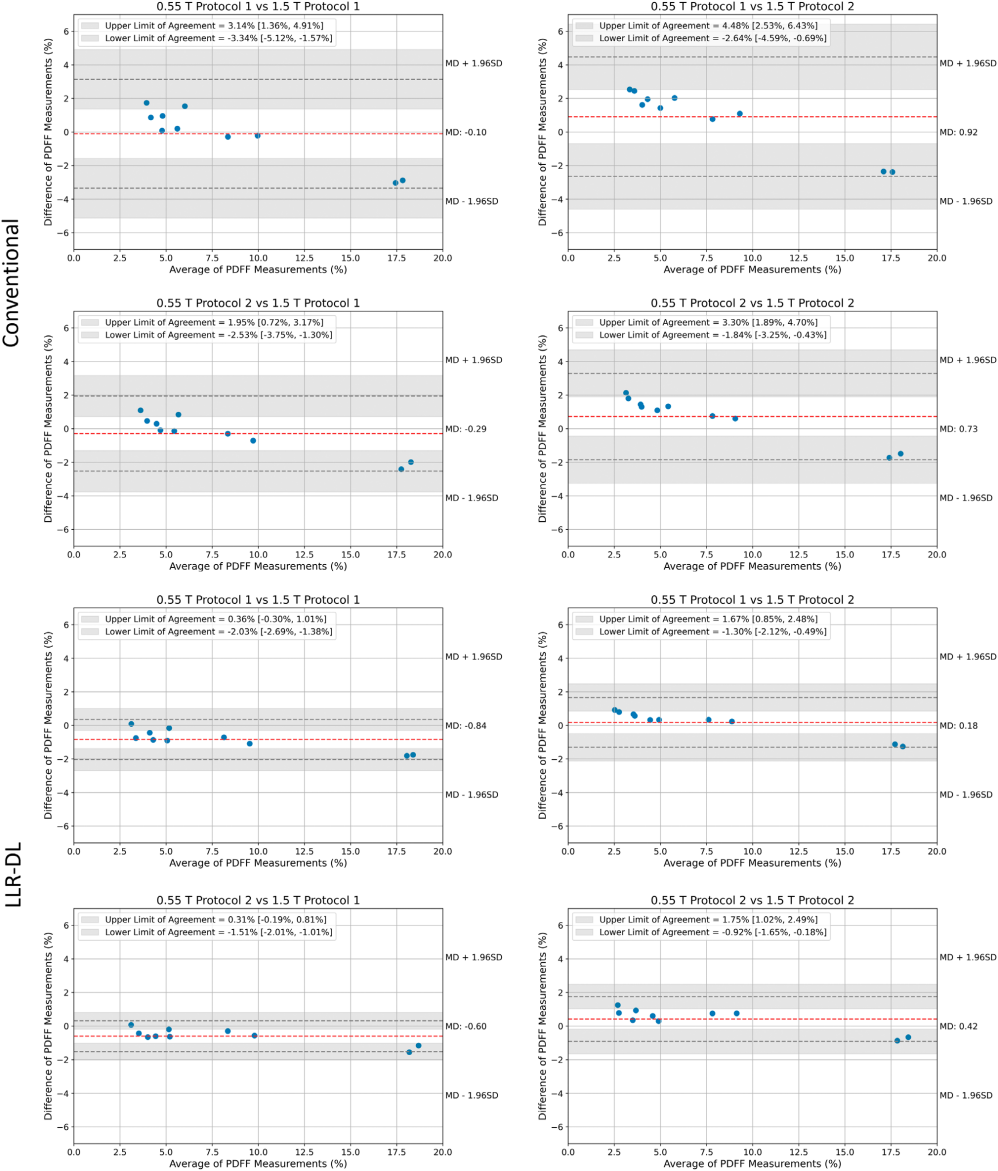
Bland–Altman plot showing the MD, LoA, and reproducibility of liver PDFF across the field strengths in vivo. Each measurement was repeated once. Volumetric analysis with liver segmentation and vessel exclusion was performed. The mean of the scan repetitions is used for Bland–Altman analysis. For LLR–DL, the repeatability coefficients in PDFF unit at 0.55 T were 0.46% for protocol 1 and 0.79% for protocol 2. At 1.5 T, the coefficients were 1.54% for protocol 1 and 0.48% for protocol 2. MD, mean difference.

The Bland–Altman plots in Figure 7 assess the 95% LoA between the 0.55 T protocols reconstructed using the conventional and LLR–DL reconstruction, compared to the 1.5 T protocols. For the conventionally reconstructed data, the mean differences range from −0.10% to 0.92%, with the upper LoA between protocols ranging from 1.95% to 4.48%, and CIs between 0.72% and 6.43%. The lower LoA ranges from −3.34% to −1.84%, with CIs between −5.12% and −0.43%. The highest variation was between protocol 1 at 0.55 T and protocol 2 at 1.5 T with a mean difference of 0.92, upper LoA of 4.48%[2.53%, 6.43%] and lower LoA −2.64%[−4.59%, −0.69%]. The best agreement was between protocol 2 at 0.55 T and protocol 1 at 1.5 T with a mean difference of −0.29, upper LoA of 1.95%[0.72%, 3.17%] and a lower LoA −2.53%[−3.75%, −1.30%].

For the LLR–DL reconstructed data, the mean differences range from −0.84% to 0.18%. The upper LoA ranges from 0.31% to 1.75%, with CIs between −0.30% and 2.49%. The lower LoA ranges from −2.03% to −0.92%, with CIs between −2.69% and −0.18%. The highest variation between the protocols on 0.55 T was again in comparison to protocol 2 on 1.5 T. Overall, the LLR–DL results show higher LoA and smaller CIs with the 1.5 T measurements, compared to the results from the conventional reconstruction.

Additionally, the repeatability coefficients at 1.5 T were 0.46% for protocol 1 and 0.45% for protocol 2. At 0.55 T, the coefficients obtained with conventional reconstruction were 1.54% for protocol 1 and 0.48% for protocol 2. When applying LLR–DL at 0.55 T, the repeatability coefficient for protocol 1 improved substantially to 0.37%, whereas protocol 2 remained unchanged at 0.48%.

## 4 DISCUSSION

In this work, two protocols with different configurations for the measurement at 0.55 T were proposed. An LLR–DL reconstruction for multi‐echo acquisition was developed to enhance image quality and improve the precision of PDFF in low‐field MRI. The conventional reconstruction produced images with low SNR for protocol 1 resulting in very noisy PDFF and $R_{2}^{*}$ maps. For protocol 2, the conventional reconstruction delivered images with sufficient SNR. However, the PDFF and $R_{2}^{*}$ maps for protocol 2 remained somewhat noisy. Compared to conventional reconstruction, LLR–DL produced images with superior SNR for protocol 1 and improved SNR for protocol 2. The increased SNR is also reflected in the PDFF and $R_{2}^{*}$ maps, leading to reduced noise‐induced bias in the PDFF values, which is most evident near to 0% and 100% PDFF.^74^ The advantages of the proposed method extend beyond its ability to jointly reconstruct multiple contrasts. It utilizes a neural network that operates on a low‐rank matrix, which decreases the complexity of the data while preserving important information about the spectral components being imaged, namely water and fat. This approach demonstrates another beneficial use of DL techniques, which have proven to highly accelerate MRI, enhance image quality, and improve patient comfort.^36^, ^75^, ^76^, ^77^

The accuracy of PDFF on low‐field was assessed by measuring an iron‐and‐fat phantom on 0.55 T and 1.5 T. The linear regression showed a statistically significant correlation between the measurements and the reference values. Pooling across all protocols and between the full range of 0%–100% PDFF, a slope of 1.00 on both protocols at 0.55 T and of 1.01 on both protocols at 1.5 T, was observed, along with the intercepts (−1.14 and −1.19 for protocols 1 and 2 at 0.55 T) and (−1.47 and −1.29 for protocols 1 and 2 at 1.5 T). These results align closely with a study performed on similar phantoms across different vendor scanners, which reported a slope range of 0.94 to 1.04 along with an intercept range of −1.53 to 2.11 PDFF.^78^ This supports the accuracy of the conducted measurement. Additionally, high precision was reported across the protocol repetitions, which indicates the consistency of the measurements over multiple trials. This is supported by the calculated repeatability coefficient of 2.33%, 2.28% for protocols 1 and 2 at 0.55 T and 1.60% and 1.84% for protocols 1 and 2 at 1.5 T, respectively. Bland–Altman plots showed a good agreement between the mean of the protocol repetitions and the reference values. The mean difference for all protocols ranges between 1.09% and 1.25%.

When using conventional reconstruction, the best LoA was observed between protocol 2 at 0.55 and protocol 1 at 1.5. This is likely due to the higher SNR when acquiring a smaller matrix compared to protocol 1 at 0.55, where some noise bias is present. The lower agreement level with protocol 2 at 1.5 T may be attributed to the fat sampling because it is acquired at in‐phase and opposed‐phase TEs and models three fat dephasing cycles, whereas the other three protocols are measured at minimum TE and model 1 to 1.5 fat dephasing cycles.

Additionally, the volunteer scans have shown the substantial advantages of using LLR–DL to reduce the bias in the PDFF values. This approach brings the PDFF measurements at 0.55 T closer to those measured at the established 1.5 T scanners, improving the comparability between the two field strengths, and ensuring the reproducibility and the precision of PDFF quantification on low‐field. LLR–DL substantially improved the repeatability of protocol 1, reducing the repeatability coefficient from 1.54% to 0.37%. This level of repeatability surpasses that of both protocols at 1.5 T, demonstrating the effectiveness of DL‐based reconstruction in enhancing measurement consistency at lower field strengths. Finally, the consistent alignment of PDFF values across field strengths further substantiates the robustness of LLR–DL. These findings indirectly validate the ability of the network to generalize to 0.55 T data despite being trained on high‐field acquisitions, further supporting the observation that such architectures generalize well when trained on large, diverse datasets, with their performance primarily driven by SNR rather than anatomical variations.^59^, ^62^

We acknowledge several limitations of this study. First, most of the volunteers have low PDFF values in the liver, which usually shows low bias in the PDFF. Here, a clinical evaluation should be performed to further ensure the precision and robustness of our method. Second, no study was conducted on the $R_{2}^{*}$ maps due to the limited sensitivity of low‐field systems to the low iron concentrations present in the volunteer data; therefore, further investigation to assess $R_{2}^{*}$ mapping, especially in cases with iron overload, should be conducted. This work does not aim to assess the clinical applicability of the method or evaluate liver iron concentration; rather, these aspects are considered directions for future work.

## 5 CONCLUSION

In this work, an LLR–DL reconstruction was proposed to reconstruct multi‐echo acquisitions on low‐field. Two protocols were proposed to perform quantitative PDFF and $R_{2}^{*}$ mapping. The robustness of our method, repeatability, and reproducibility of PDFF quantification on 0.55 T systems were assessed in comparison to PDFF quantification on 1.5 T systems on both a phantom and volunteers. The results demonstrate that the use of the precalibrated nine‐peak fat spectrum model enables accurate and consistent PDFF estimation across field strengths, without the need for field‐specific recalibration.

## CONFLICT OF INTEREST STATEMENT

Majd Helo receives PhD funding from Siemens Healthineers AG. Dominik Nickel and Stephan Kannengiesser are employed by Siemens Healthineers AG.

## Supporting information


**DATA S1.** Supporting Figures.

## Data Availability

The data used in this study are not publicly available.
