# Supplementary material for: Liver fat quantification at 0.55 T enabled by locally low‐rank enforced deep learning reconstruction
Source: Magn Reson Med. 2025 Aug 29;95(1):569–84. doi: 10.1002/mrm.70057 (PMC12620156; doi:10.1002/mrm.70057)
Supplement: Supplementary file 1 — DATA S1. Supporting Figures. [file MRM-95-569-s001.docx]

# Supplementary Information


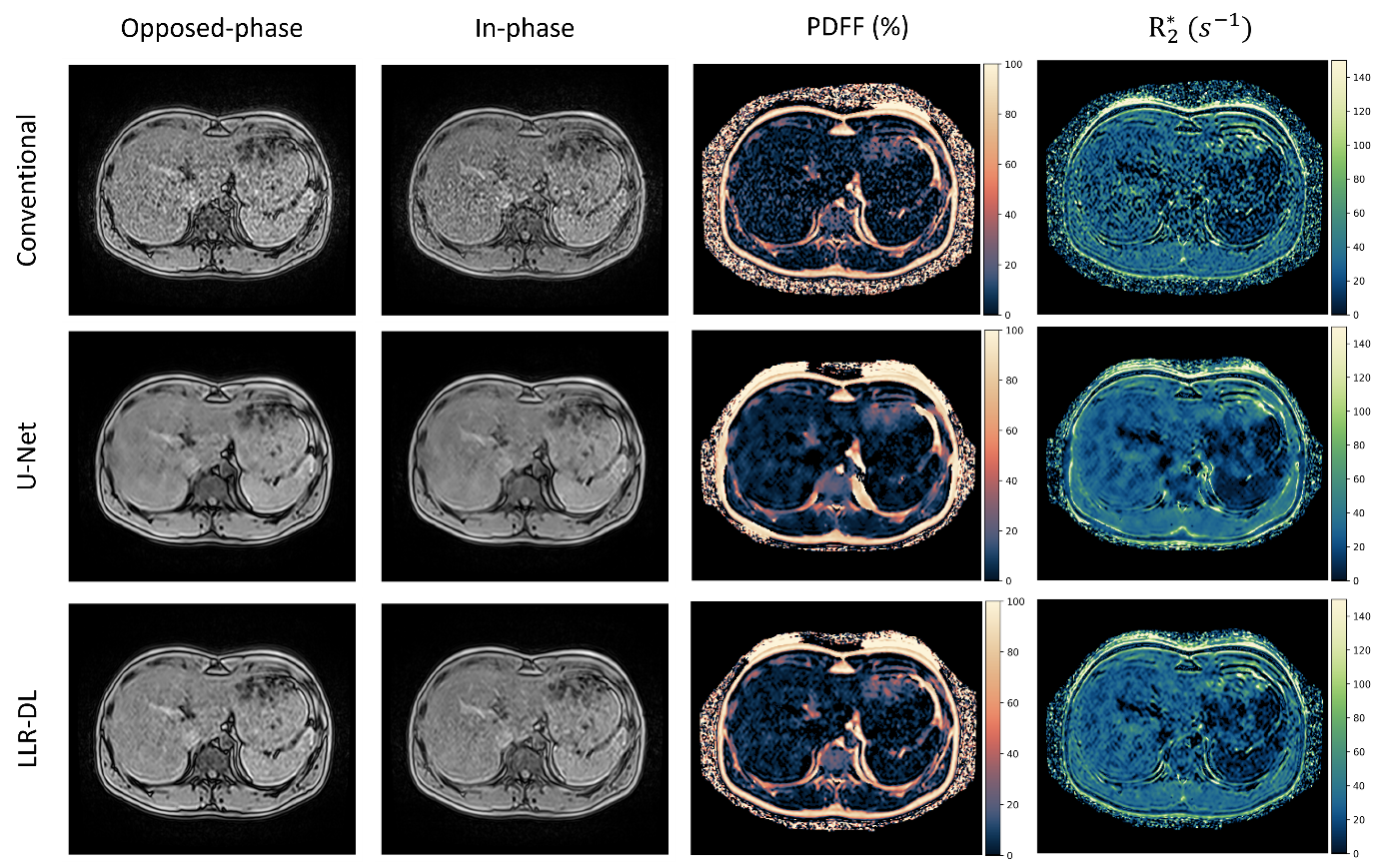


Supplementary Figure S1: Reconstruction results of opposed- and in-phase images at low-field corresponding to echoes 3 and 6 in Protocol 1. The proposed LLR-DL method demonstrates superior image quality compared to both the conventional CAIPIRINHA reconstruction and an image-based 3D U-Net. The 3D U-Net tends to oversmooth the anatomical structures. For all reconstruction results, the SNR level is visible in the fitted quantitative PDFF and R2* Maps. Images are shown for a volunteer with 4.5% liver PDFF.


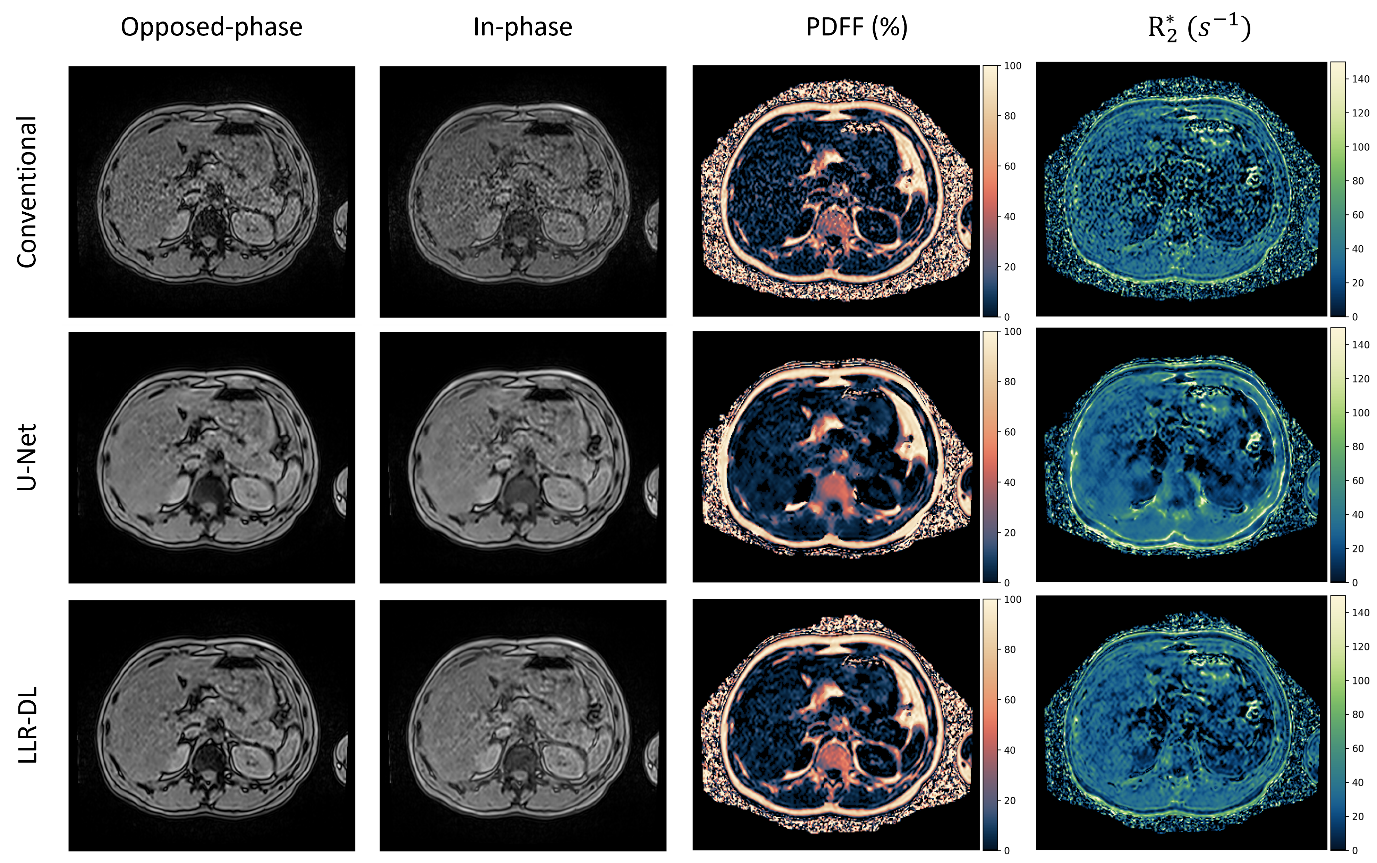


Supplementary Figure S2: Reconstruction results of opposed- and in-phase images at low-field corresponding to echoes 3 and 6 in Protocol 1. The proposed LLR-DL method demonstrates superior image quality compared to both the conventional CAIPIRINHA reconstruction and an image-based 3D U-Net. The 3D U-Net tends to oversmooth the anatomical structures. For all reconstruction results, the SNR level is visible in the fitted quantitative PDFF and R2* Maps. Images are shown for a volunteer with 5.0% liver PDFF.


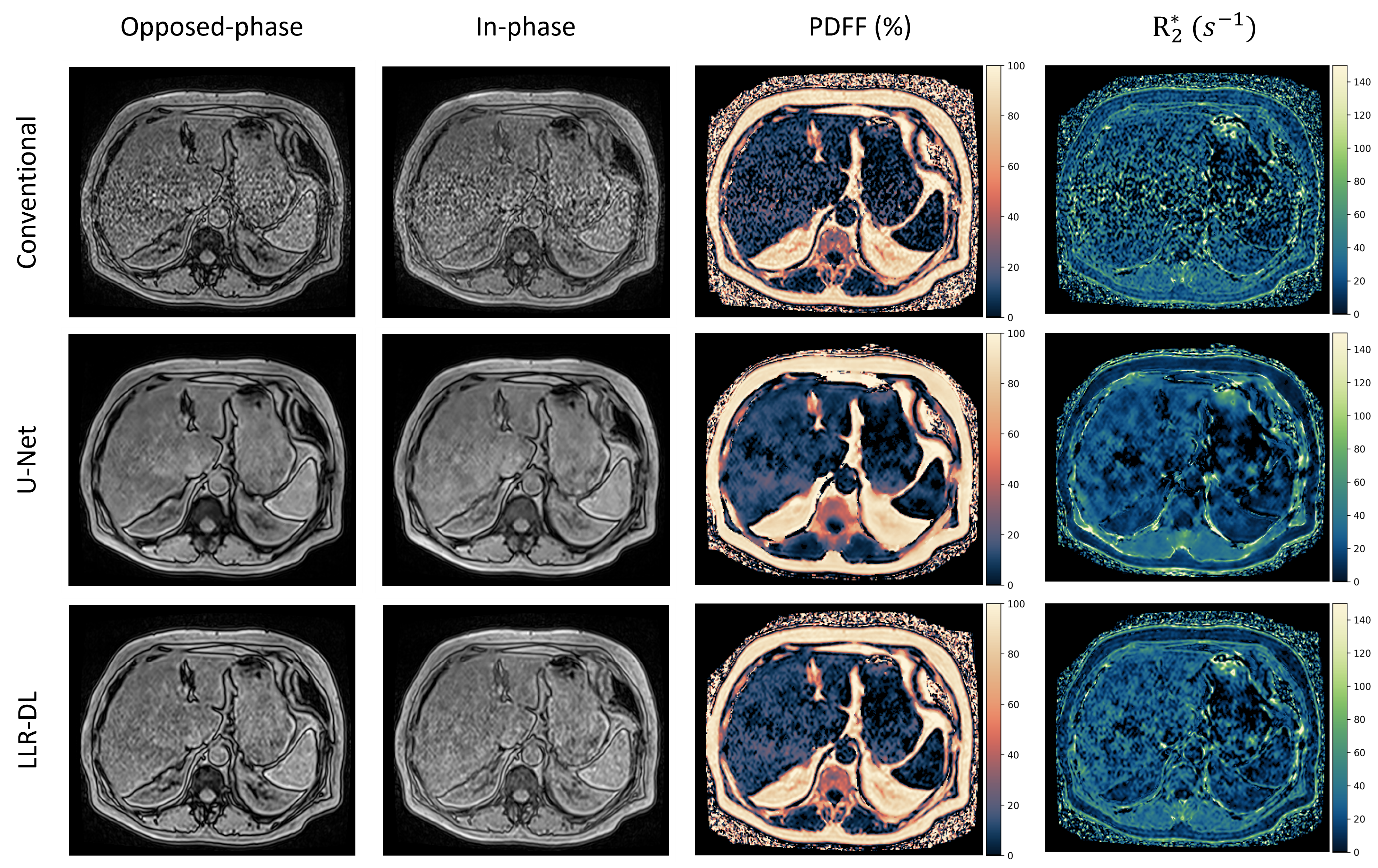


Supplementary Figure S3: Reconstruction results of opposed- and in-phase images at low-field corresponding to echoes 3 and 6 in Protocol 1. The proposed LLR-DL method demonstrates superior image quality compared to both the conventional CAIPIRINHA reconstruction and an image-based 3D U-Net. The 3D U-Net tends to oversmooth the anatomical structures. For all reconstruction results, the SNR level is visible in the fitted quantitative PDFF and R2* Maps. Images are shown for a volunteer with 10% liver PDFF.


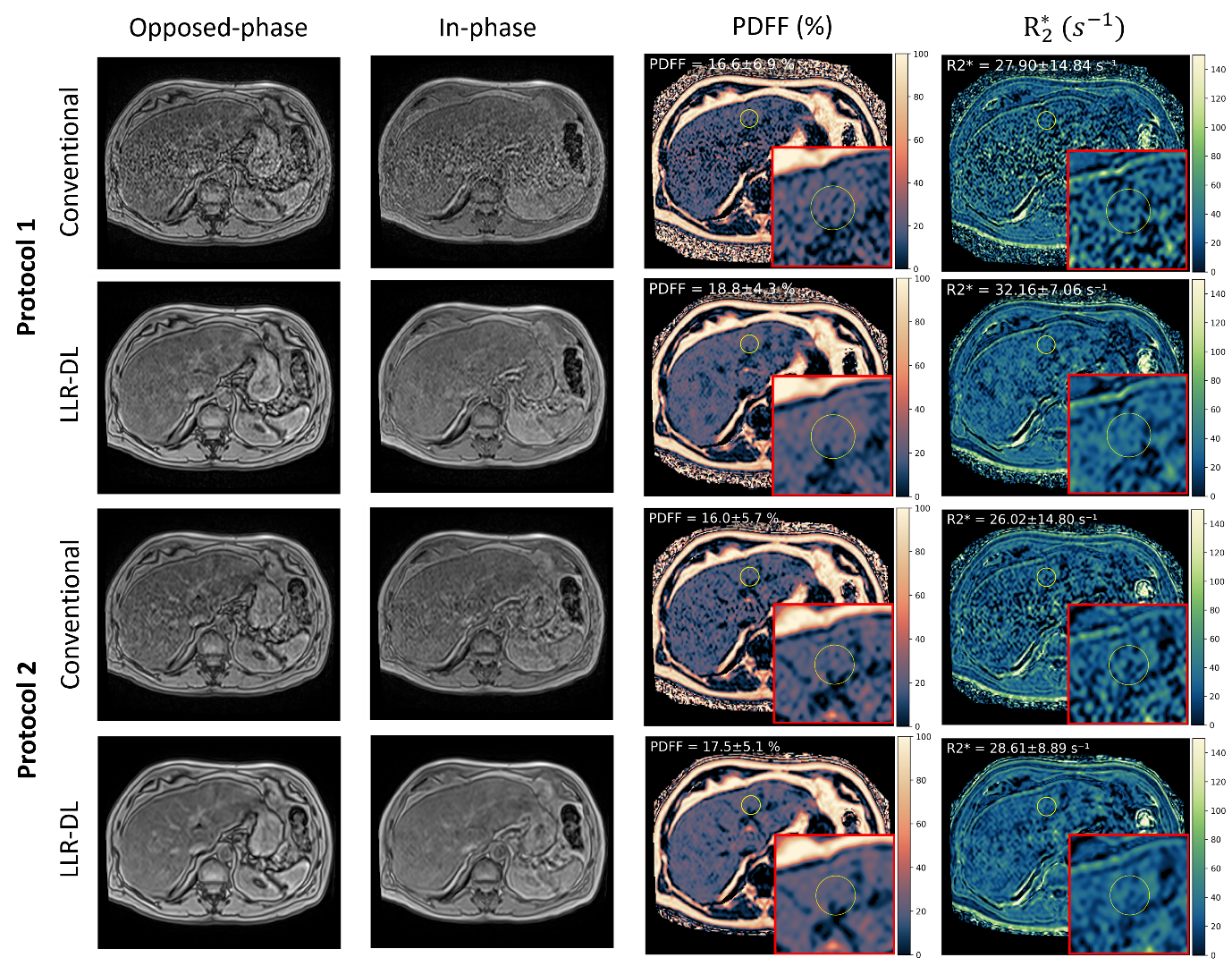


Supplementary Figure S4: Reconstruction results of opposed- and in-phase images at low-field corresponding to echoes 3 and 6 in both protocols. The upper two rows are the reconstruction results of the data acquired using protocol 1. The proposed LLR-DL shows superior image quality to the conventional CAIPIRINHA reconstruction. The lower two rows show the reconstruction results of protocol 2. Here, the conventional CAIPIRINHA method provides images with good SNR, the LLR-DL however enhanced the SNR. For all reconstruction results, the SNR level is visible in the fitted quantitative PDFF and R2* Maps. An example ROI is drawn in the liver on the quantitative maps with a zoomed-out view. The corresponding mean and standard deviation are reported.


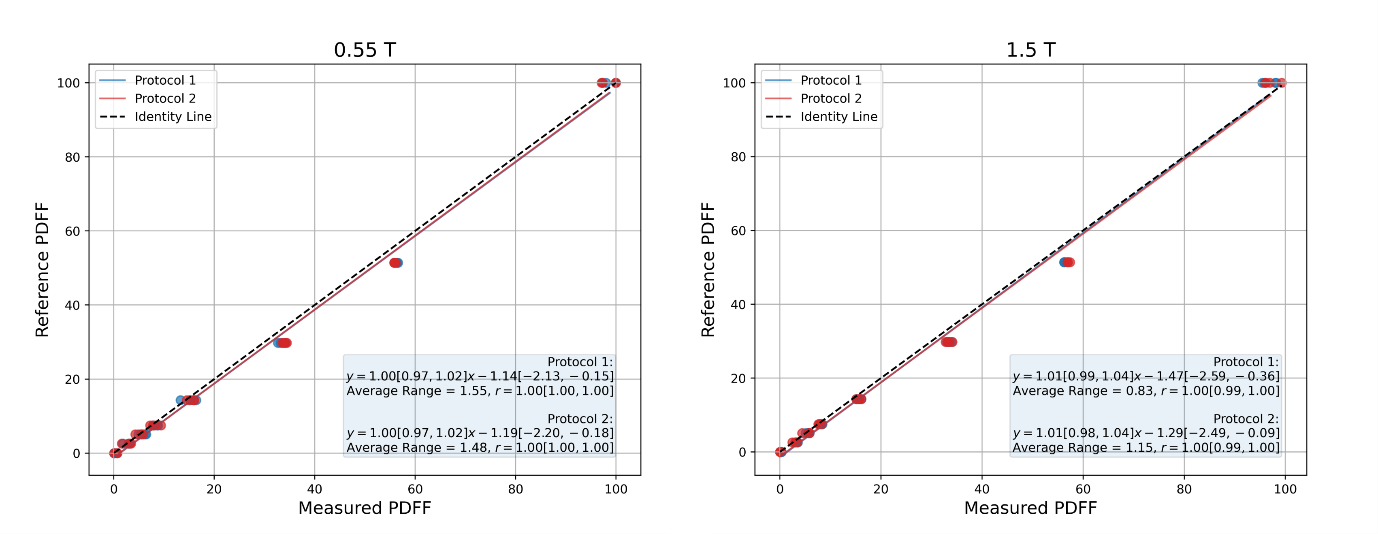


Supplementary Figure S5: Linear regression plots comparing ROI based MRI-PDFF measurements with the reference values from the phantom datasheet. The dashed line represents the identity between the measurements and nominal values. For each protocol, the slopes, intercepts of the regression lines, and Pearson’s correlation coefficients r (with 95% confidence intervals) are depicted.
